# Organization of Tennessee Dental Association

**Published:** 1867-11

**Authors:** Wm. T. Arrington

**Affiliations:** Rec. Sec.


### CORRESPONDENCE.

---

### *Organization of Tennessee Dental Association.*

Pursuant to a call made at a preliminary meeting of the Dentists of Memphis on the 20th of June, the following members of the profession convened at Nashville on the 26th of July and organized the *Tennessee Dental Association.*

Present—Drs. Wm. H. Morgan, Nashville; G. W. Acree, Memphis; Wm. T. Arrington, Memphis; J. B. Wasson, Memphis; J. A. Arrington, Jackson; H. M. Acree, Clarksville; R. Russell, Nashville; J. C. Ross, Nashville; S. J. Cobb, Nashville; W. P. Wilson, Nashville; Alex. Hartman, Murfreesboro'; M. McCarty, Pulaski; T. E. Beech, Franklin; W. R. Johnston, Columbia.

At 10 o'clock A. M. with Dr. W. H. Morgan as chairman pro. tem., and Dr. Ross, Sec., the house was called to order, and the following committee appointed to draft Constitution and By-Laws—Dr. Wm. T. Arrington, Chairman; Dr. S. J. Cobb, Dr. W. R. Johnston.

A Nominating Committee was also appointed—Dr. J. B. Wasson, chairman; Dr. Alex. Hartman, Dr. R. Russell.

While the committee were in session Drs. G. W. Acree of Memphis and W. H. Morgan of Nashville addressed the meeting upon the subject of *Dental Education, State and Local Organization*, and the importance of prompt and immediate action towards the general advancement of the dental profession.

Committee on Constitution and By-Laws presented a paper which was received, and the committee discharged.

On motion the paper was taken up by section, discussed, voted upon and approved, then voted and approved as a whole, and adopted as the Constitution and By-Laws under which to organize.

Committee on Nomination made the following report which was received—Dr. W. H. Morgan for *President*; Dr. J. B. Wasson, 1*st Vice President;* Dr. J. C. Ross, 2*nd Vice President;* Dr. Wm. S. Arrington, *Rec. Sec.;* Dr. R. Russell, *Cor. Sec.;* Dr. Alex. Hartman, *Treas.*; *Ex. Committee;* Dr. G. W. Acree, Dr. J. A. Arrington, Dr. W. R. Johnston. Meeting then adjourned, and convened at 2½ P. M.

An election was held and those nominated duly elected by ballot to serve as officers of the association for the term of one year, and after being properly installed the President made a few appropriate remarks, and then declared the Association organized and ready for business.

On motion of Dr. Arrington it was resolved that the *American Code of Dental Ethics* be approved and adopted by the Association. Adjourned to meet to-morrow morning at 9 o'clock.

SATURDAY, *July 27th.*

Society met, minutes read and approved. On motion it was resolved that the Semi-Annual meeting be held at Jackson from the 20th to the 24th of next December, and the Annual Meeting to be held in Memphis, on Wednesday, Thursday, Friday and Saturday preceeding the last Tuesday in July, 1868.

Drs. Morgan, Acree and Beech were duly elected delegates to the American Dental Association at Cincinnati.

Many interesting subjects were discussed, and the liveliest interest manifested by all present.

It was resolved that the Secretary be requested to furnish the *American Journal of Dental Science, The Dental Cosmos*, and the *Dental Register* with a synopsis of the proceedings.

Adjourned at 2 P. M. to meet at Jackson on the 20th of December next.

WM. T. ARRINGTON, *Rec. Sec.*

